# Generative AI/LLMs for Plain Language Medical Information for Patients, Caregivers and General Public: Opportunities, Risks and Ethics

**DOI:** 10.2147/PPA.S527922

**Published:** 2025-07-31

**Authors:** Avishek Pal, Tenzin Wangmo, Trishna Bharadia, Mithi Ahmed-Richards, Mayank Bhailalbhai Bhanderi, Rohitbhai Kachhadiya, Samuel S Allemann, Bernice Simone Elger

**Affiliations:** 1 Institute for Biomedical Ethics, University of Basel, Basel, Switzerland; 2 Patient Author, The Spark Global, Buckinghamshire, UK; 3 Centre for Pharmaceutical Medicine Research, King’s College London, London, UK; 4 Current Medical Research & Opinion, Taylor & Francis Group, London, UK; 5 Patient Author, Scleroderma and Raynauds UK, London, United Kingdom; 6 Innomagine Consulting Private Limited, Hyderabad, India; 7 Department of Pharmaceutical Sciences, University of Basel, Basel, Switzerland; 8 Center for Legal Medicine, University of Geneva, Geneva, Switzerland

**Keywords:** artificial intelligence, large language model, ethics, health literacy, plain language summary

## Abstract

Generative artificial intelligence (gAI) tools and large language models (LLMs) are gaining popularity among non-specialist audiences (patients, caregivers, and the general public) as a source of plain language medical information. AI-based models have the potential to act as a convenient, customizable and easy-to-access source of information that can improve patients’ self-care and health literacy and enable greater engagement with clinicians. However, serious negative outcomes could occur if these tools fail to provide reliable, relevant and understandable medical information. Herein, we review published findings on opportunities and risks associated with such use of gAI/LLMs. We reviewed 44 articles published between January 2023 and July 2024. From the included articles, we find a focus on readability and accuracy; however, only three studies involved actual patients. Responses were reported to be reasonably accurate and sufficiently readable and detailed. The most commonly reported risks were oversimplification, over-generalization, lower accuracy in response to complex questions, and lack of transparency regarding information sources. There are ethical concerns that overreliance/unsupervised reliance on gAI/LLMs could lead to the “humanizing” of these models and pose a risk to patient health equity, inclusiveness and data privacy. For these technologies to be truly transformative, they must become more transparent, have appropriate governance and monitoring, and incorporate feedback from healthcare professionals (HCPs), patients, and other experts. Uptake of these technologies will also need education and awareness among non-specialist audiences around their optimal use as sources of plain language medical information.

## Plain language summary

More and more people are using special computer programs called artificial intelligence (AI) or large language models (LLMs) to find and get medical facts in simple words they can understand. This can help people take better care of themselves, learn about their health, and talk with their doctors. We found that AI/LLMs generally provided correct and helpful information to people. However, there could also be a risk of incorrect or unreliable information in certain situations if the question is complex. This can cause harm to people if they use this information to make their own medical decisions. Also, gAI/LLMs provide human-like responses, which make people trust them more than they should. There could be a risk that people may share their medical information with AI/LLMs, which could get into the wrong hands. To make sure these programs really help people, they need to be clear about how they work, and they must have good rules to follow and take advice from doctors and patients to improve performance. People also need to be trained on how to best use these AI tools to find easy-to-understand and reliable medical information. It is important for doctors, patients and other health workers to help make sure the AI is producing reliable and understandable medical information for patients.

## Introduction

In recent years, there has been widespread use of generative artificial intelligence (gAI) and large language models (LLMs) in healthcare, including improving clinical decision-making, clinical documentation, operational efficiency, diagnostic support, patient monitoring and follow-ups, healthcare professional (HCP) education and much more.^1–3^ The attraction of AI applications in healthcare can be gauged from the significant increase in the number of annual authorizations of AI medical devices by the US Food and Drug Administration (FDA) from a mere two authorizations in 2016 to 69 in 2022.^4^ Interestingly, a number of AI health tools available in the market are, in fact, not validated based on actual clinical data. This already raises the fundamental ethical question of whether the output from these models could pose a risk to patients when implemented in healthcare settings.

Patients, caregivers, and the general public (henceforth to be called non-specialist audiences) are using gAI/LLMs, such as ChatGPT, Google Bard, LLaMa, and NVLM, to access medical information and to simplify and/or translate complex medical terminology into plain everyday language through conversational interfaces. Their questions may include information about medical conditions, the latest medical research, and treatment options, including lifestyle modifications.^5–7^ Their ultimate aim most likely is to increase their knowledge and understanding of a diagnosis or treatment and, by extension, improve their health literacy, defined as the ability to find, understand and use health information to make informed decisions.^1^,^3^,^8^ There are two primary reasons for the unprecedented popularity of gAI/LLMs among the general public compared with previous AI technologies. First, they offer mostly free access to easy-to-understand (plain language), summarized content, and, more importantly, ease of use without requiring knowledge of programming languages or coding.^9^ However, such accessibility also poses deeper ethical questions, such as the impact on autonomy and/or active participation in shared decision-making on self-care or care for their families. Acknowledging the increasing demand for medical information to be more accessible and understandable to non-specialist audiences, organizations have started to utilize AI-based models to automate the unsupervised development and access to plain language medical information for non-specialist audiences.^10^ It can be foreseen that gAI/LLMs will continue to grow as a tool used by organizations to provide medical information. However, with increased accessibility comes the risk that such users may use gAI/LLMs as a single source of truth and implement changes in their disease management, lifestyle, and disease prevention, which could have harmful effects on their health.^11^ Hence, appropriate ethical governance and monitoring of these models require serious deliberation considering their growing application in healthcare in general and, more specifically, in patient education.

While the use of gAI/LLMs by non-specialist audiences has been increasing, the majority of research has continued to focus on the use of gAI/LLMs by HCPs/researchers for disease diagnosis, clinical/patient note generation or other HCP-monitored activities.^12–15^ The next most frequent line of enquiry has been on how institutions are using gAI/LLMs to bring efficiency into healthcare delivery.^16^,^17^ Consequently, published ethical explorations have also focused on these two broad themes.^18–20^ This leaves a concerning unaddressed gap regarding the risks, opportunities and ethical considerations when non-specialist audiences use gAI/LLMs as sources of plain language medical information. To our knowledge, this is the first attempt to start investigating this knowledge gap. Our aims were three-fold, namely to: (1) provide an overview of findings in published primary research on the applications, limitations, risks, and potential harms associated with the utilization of gAI/LLMs in educating non-specialist audiences (2) critically build on the ethical perspective on the use of gAI/LLMs by this non-specialist audience, and (3) make recommendations, including those specifically from patients, for a balanced approach to the implementation of gAI/LLMs.

## Methods

We used the framework of Arksey and O’Malley^21^ to identify and select eligible articles and collated and summarized the results. Salient papers were identified by means of a structured search of Ovid, using a search string that captured the relationship between synonyms of LLM and AI and medical information for patients. The following search string was used: guidance OR guideline OR recommendations OR regulations AND plain language materials OR plain language resources OR patient materials OR lay summaries OR plain language summaries OR plain English summaries OR non-technical summaries OR patient summaries. We restricted our search to open-access publications dating from January 2023 to July 2024 to allow sufficient time for literature to accrue following the rollout of the most prominent gAI/LLMs, ChatGPT, in November 2022, followed by Copilot and LlaMa in February 2023, Gemini and Claude in March 2023, and Mistral in April 2023. No geographical restrictions were imposed.

Papers that reported outcomes of comparisons across various gAI/LLMs or between gAI/LLMs and other information sources (eg, Google, clinical guidelines, medical society recommendations or patient organization materials) were included. We excluded papers that were in languages other than English and those that reported outcomes of assessments of gAI/LLMs for any other uses beyond sources of medical information for patients (eg, assessment of utilization by HCPs as aids in patient care decision-making, predictive diagnosis, data analysis, medical procedures, or administrative tasks such as patient record management). We also excluded any reviews, commentaries, or any article types that did not report primary data.

The titles and abstracts of articles were screened independently by two researchers (AP, supported by AY as noted in our acknowledgements) to confirm that they met the inclusion criteria and to eliminate duplicates. Full-text articles were then independently assessed for eligibility by three researchers (AP, and MBB and RK together), who also approached the extraction and cross-check steps in a similar independent manner. Disagreement was resolved through discussion. The following fields were extracted from the included articles: study objectives, disease area or procedure, LLMs assessed, the purpose of use of LLMs, evaluation criteria, evaluation method, what the models did and did not do well, overall risks, and specific patient feedback, if any.

During the information extraction stage, we noted that none of the articles included in our assessment discussed the topic of ethical considerations around non-specialist audiences using gAI/LLMs as the source of medical information. In order to address this crucial knowledge gap, we performed a supplementary review of the literature. The intention was not to perform a comprehensive review but rather a snapshot of the relevant and latest publications on this topic to initiate a conversation that has been mostly missing in public discourse.

## Results

### Characteristics of Articles and Nature of Analyses

Overall, 918 journal articles were identified; of these, 51 met the eligibility criteria. Open-access versions were not available for five journal articles, while two studies were not primary research, and hence, these were excluded. Finally, 44 journal articles were included where gAI/LLMs were evaluated as sources of plain language medical information for patients.

Tables 1 and 2 and Supplementary Tables S1 and S2 provide an overview of the various investigations performed to assess the utility of gAI/LLMs evaluated as sources of plain language medical information for patients. These investigations either (1) evaluated a single model such as ChatGPT or Bard or Claude or Bing (Table 1); or (2) compared models to patient materials or patient guidelines (Table 2); or (3) compared different models (Supplementary Table S1); or (4) compared models to search engines or an AI app’s recommendations to expert advice (Supplementary Table S2).

**Table 1:** Studies Evaluating LLMs

Publication	Study Objective(s)	Disease Area or Procedure	LLM Assessed	LLM Use	Evaluation Criteria	Evaluation Method
Polat 2024^22^	To investigate the potential of the ChatGPT as a parental information tool on pediatric Adenoidectomy, tonsillectomy, and ventilation tube insertion surgery (ATVtis)	ATVtis surgery	ChatGPT	To identify the top 15 FAQs by parents for ATVtis surgical procedures	Accuracy, readability	Grading scale to measure accuracy, FRE, FKGL
Sarraju 2023^23^	To evaluate the appropriateness of AI model responses to simple, fundamental CVD prevention questions	Cardiovascular disease prevention	ChatGPT	To answer questions addressing fundamental preventive concepts, including risk factor counseling, test results, and medication information	Appropriateness	Classification as “appropriate” or “inappropriate”
Coban 2024^24^	To evaluate the quality of patient information by assessing the responses of the ChatGPT model to questions related to medication-related osteonecrosis of the jaw	Medication-related osteonecrosis of the jaw	ChatGPT	To answer questions related to medication-related osteonecrosis of the jaw	Quality	GQS
Biswas 2023^25^	To evaluate the accuracy of ChatGPT in providing accurate and quality information to answer questions on myopia	Myopia	ChatGPT	To answer common questions that patients typically ask	Accuracy, quality	Likert scale
Balel 2023^26^	To assess the usability of the information generated by ChatGPT in oral and maxillofacial surgery	Oral and maxillofacial surgery	ChatGPT	To answer common questions asked by patients about oral and maxillofacial surgery procedures.	Quality	Modified GQS
Ghanem 2024^27^	To evaluate the accuracy of ChatGPT in delivering evidence-based information related to osteoporosis	Osteoporosis	ChatGPT	Twenty of the most common FAQs related to osteoporosis were subcategorized into diagnosis, diagnostic method, risk factors, and treatment and prevention	Accuracy	Scale from 0 (harmful) to 4 (excellent)
Nielsen 2023^28^	To evaluate accuracy, relevance, and depth of patient information provided by ChatGPT concerning prevalent otolaryngologic conditions	Otolaryngology	ChatGPT	To answer common questions that patients typically ask	Accuracy, depth of information, relevance	Likert scale
Seth 2023^29^	To investigate whether ChatGPT-4 could provide safe and up-to-date medical information about breast augmentation that is comparable to other patient information sources	Plastic surgery	ChatGPT	To answer common questions that patients typically ask	Accuracy, accessibility, informativeness	Qualitative
Floyd 2024^30^	To evaluate the accuracy and comprehensiveness of ChatGPT in radiation oncology-related domains	Radiation oncology	ChatGPT	To answer common questions that patients typically ask. To answer 40 questions related to landmark studies. To answer questions requesting literature review	Accuracy, comprehensiveness	Point based scoring
Keysser 2024^31^	To find out whether ChatGPT is able to provide qualified answers on the applicability of complementary and alternative medicine methods for rheumatoid arthritis, systemic lupus erythematosus, and granulomatosis with polyangiitis	Rheumatology	ChatGPT	To advise Complementary and alternative medicine treatment	Reliability	Likert scale
Valentini 2024^32^	To evaluate ChatGPT’s answers to sarcoma-related inquiries for completeness, misleading content, accuracy, appropriateness, currency	Sarcoma	ChatGPT	To answer sarcoma-related questions	Accuracy, appropriateness, completeness, currency, misleadingness	Likert scale
Rasmussen 2023^33^	To evaluate the accuracy of responses to typical patient-related questions on vernal keratoconjunctivitis	Vernal keratoconjunctivitis	ChatGPT	To answer common questions that patients typically ask	Quality	Likert scale

**Abbreviations**: AI, artificial intelligence; FAQ, frequently asked question; GQS, Global Quality Scale; LLM large language model; FKGL, Flesch-Kincaid Grade Level; FRE(S), Flesch Reading Ease (Score).

**Table 2:** Studies Comparing LLMs with Current Standards (Eg, Established Healthcare Information or HCP Guidelines)

Publication	Study Objective(s)	Disease Area or Procedure	Comparators	LLM Use	Evaluation Criteria	Evaluation Method
Rahimli Ocakoglu 2024^51^	To evaluate the accuracy, completeness, precision, and readability of outputs generated by three large language models	Pelvic organ prolapse	ChatGPT vs Bard vs Bing vs patient information material	To answer common questions that patients typically ask	Accuracy, completeness, precision, readability	SMOG, FKGL
Stroop 2023^34^	To evaluate the validity of a LLM in providing medical information	Spinal surgery (acute lumbar disc herniation)	ChatGPT vs standard informed consent form	To get the clinical picture of acute lumbar disc herniation	Accuracy, comprehensiveness, ease of understanding, specificity, validity, empathy	Survey response
Citron 2023^35^	To assess the safety and accuracy of the responses to questions that may be posed by patients exploring aesthetic surgery	Aesthetic surgery	ChatGPT vs Bard vs Bing vs criteria from the NHS website	To answer “How should I choose my aesthetic surgeon in the UK”. To recommend surgeons for three common aesthetic procedures: breast augmentation, rhinoplasty, and abdominoplasty. To answer whether a specifically named surgeon was “good” and “summarize complications associated with three common cosmetic procedures”	Accuracy, safety	NA
Currie 2023^36^	To evaluate the capabilities of ChatGPT for generating patient information sheets suitable for use in gaining informed consent.	Nuclear Medicine	ChatGPT vs patient information material	To generate patient information sheets suitable for use in gaining informed consent	Accuracy, appropriateness, currency, fitness for purpose	Category: “Poor”, “Below average”, “Average”, “Above average”
Sciberras 2024^37^	To assess the reliability of responses generated by ChatGPT for a set of frequently asked questions posed by patients with inflammatory bowel disease	Inflammatory bowel disease	ChatGPT vs ECCO guideline	To answer common questions that patients typically ask (questions framed by patient representative)	Accuracy	Likert scale
Szczesniewski 2023^38^	To assess the quality of the information provided by AI like ChatGPT and establish if it is a secure source of information for patients	Urological diseases	ChatGPT vs EAU clinical guidelines	To answer questions about pathology and general treatment.	Quality	DISCERN
Gabriel 2023^39^	To assess the ChatGPT artificial intelligence platform’s utility and accuracy as a patient education tool in robotic-assisted radical prostatectomy	Robotic radical prostatectomy	ChatGPT vs patient information material	To answer common questions that patients typically ask	Accuracy, relevance	Qualitative
Walker 2023^40^	To assess the reliability of medical information provided by ChatGPT in hepato-pancreatico-biliary conditions.	HPB conditions	ChatGPT vs clinical guidelines and static internet	To answer common questions that patients typically ask	Reliability	EQIP
Cappellani 2024^41^	To assess the accuracy of ophthalmic information provided by an AI chatbot	Ophthalmic disease	ChatGPT vs AAO guidelines	To find information about what X is and how X is diagnosed and treated	Accuracy, reliability	Scores ranging from −3 (unvalidated and potentially harmful to a patient’s health or well-being if they pursue such a suggestion) to 2 (correct and complete)
Casciato 2024^42^	To characterize the quality and readability of foot and ankle pathology-specific responses to common queries	Foot and Ankle Surgery	ChatGPT vs FootCareMD	To answer FAQs concerning foot and ankle surgeries.	Quality, readability	FRES, FKGL, DISCERN
Roldan‐Vasquez 2024^43^	To evaluate the accuracy, comprehensiveness, and reliability of ChatGPT’s responses to the questions asked by patients about breast cancer surgery	Breast surgical oncology	Chat GPT vs patient information material	To answer common questions that patients typically ask	Accuracy, comprehensiveness, reliability	PEMAT
Janopaul-Naylor 2024^44^	To assess the quality of responses to common questions for patients with cancer	Cancer	ChatGPT, Bing vs patient information material	To answer common questions that patients typically ask	Quality	DISCERN
Verran 2024^45^	To determine whether AI can produce patient information leaflets that include a similar degree of content to current British Association of Dermatologists PILs	Dermatology	ChatGPT vs patient information material	To generate patient information leaflet	Completeness, readability	FRET, FKGL
Abou-Abdallah 2024^46^	To assess the quality and readability of surgical procedural information provided by ChatGPT and compare this with established healthcare information from ENT UK	ENT operations	ChatGPT vs Established healthcare information	The questions posed to ChatGPT were: “Tell me about having a tonsillectomy”, “Tell me about having an adenoidectomy” and “Tell me about having grommet surgery”	Quality, readability	FRES, FKGL, GFI, SMOG, DISCERN
Halawani 2024^47^	To compare the readability and accuracy of large language model generated patient information materials to those supplied by the American Urological Association, Canadian Urological Association, and European Association of Urology for kidney stones	Kidney stone	ChatGPT vs patient information material	To answer the most frequent patient questions related to kidney stones	Accuracy, readability	Likert scale, SMOG, GFI, FKGL
Lopez-Ubeda 2024^48^	To compare different LLM-based approaches for automatic summary generation in radiology	Knee MRI reports	T5 (Text-to-Text Transfer Transformer), BART, RNN vs Radiologist	Compare knee MRI summaries generated by various LLM as well as radiologists	Accuracy, coherence, consistency, fluency, relevance	SummEval benchmark, BLEU, METEOR, Rouge-L
Lockie 2024^49^	To evaluate a Chat GPT-generated patient information leaflet against a surgeon-generated version in order to explore a potential application of this AI language processing model.	Laparoscopic cholecystectomy	ChatGPT vs HCP	To generate patient information leaflet. Patient-assessed quality of patient information leaflet	Quality	Questionnaire
Coskun 2023^50^	To evaluate the performance of ChatGPT in providing patient information on prostate cancer and to compare the accuracy, similarity, and quality of the information to a reference source	Prostate cancer	ChatGPT vs patient information material	To answer of common questions that patients typically ask	Accuracy, precision, quality, recall	GQS

**Notes**: ^a^Involved actual patients in the study.

**Abbreviations**: AAO, American Academy of Ophthalmology; AI, artificial intelligence; BART, Bidirectional and Auto-Regressive Transformers; BLEU, Bilingual Evaluation Understudy; EAU, European Association of Urology; ENT, ear nose and throat; EQIP, Ensuring Quality Information for Patients; FKGL, Flesch-Kincaid Grade Level; FRE(S), Flesch Reading Ease (Score); GFI, Gunning Fog Index; GQS, Global Quality Scale; HCP, healthcare professional; HPB, hepato-pancreatico-biliary; LLM, large language model; METEOR, Metric for Evaluation of Translation with Explicit Ordering; MRI, magnetic resonance imaging; NHS, National Healthcare Service; PEMAT(-P), Patient Education Material Assessment Tool (Printable); PIL, patient information leaflet; RNN, Recurrent Neural Network; SMOG, Simplified Measure of Gobbledygook.

Disease areas or specialties explored most frequently included different types of cancer, cardiovascular disease, ophthalmological disorders, rheumatology, dermatology, otolaryngology, and surgery. The objectives included the evaluation of various attributes such as accuracy, readability, appropriateness, quality, comprehensiveness, relevance, reliability, precision, accessibility, actionability, and empathy. Irrespective of the terminology used in the study objectives, the majority of the assessments were based on the clinical judgement of HCPs. A similar approach was used to assess the appropriateness, relevance, quality, precision, reliability, accessibility, or actionability of the content generated. Readability and comprehension were assessed by a variety of scales, including the Flesch-Kincaid Grade Level, Flesch Reading Ease Score, Simple Measure of Gobbledygook, or Gunning Fog Index. Quality was evaluated based on the Global Quality Scale, which is a standard for assessing the quality of online resources or by medical experts based on their clinical experience/judgement or using the DISCERN scale or Patient Education Materials Assessment Tool. While all the studies aimed to provide patient perspectives, only three of them involved actual patients; this involved seeking feedback from patients/patient representatives on commonly asked questions or on an information leaflet generated by ChatGPT or about following an AI-advised exercise regimen in plain language, without medical supervision.

### Overview of the Performance of gAI/LLMs

**Figure 1:**
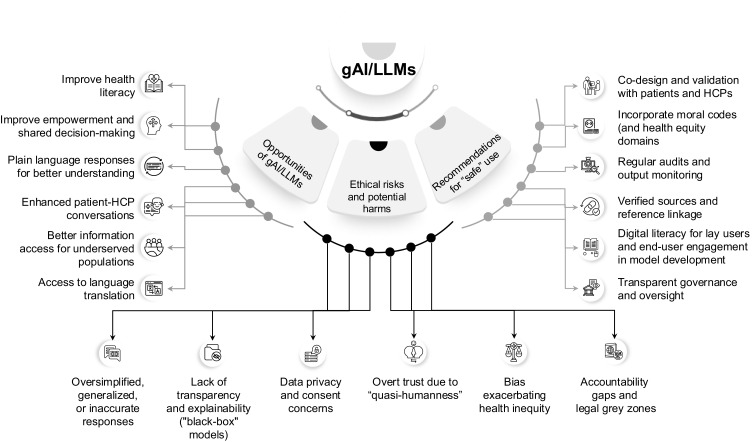
The opportunities, risks and recommendations for the use of gAI/LLMs for plain language medical information for non-specialist audiences.

**Abbreviations**: gAI, generative artificial intelligence; HCP, healthcare professional; LLM large language model.

Figure 1 and Table 3 provide an overview of what gAI/LLMs reportedly did well, areas for improvement, overall risks, and specific patient feedback when evaluated as a source of plain language medical information for patients. In investigations of individual models (Table 1), comparative studies across models (Supplementary Table S1), models versus patient materials (Table 2) or internet search engines (Supplementary Table S2), the majority of the models provided reasonably helpful, accurate and well-balanced responses that required minimal clarification and were up-to-date based on treatment guidelines or HCP recommendations. The responses generated were grammatically accurate, sufficiently readable, appropriately detailed, and patient-oriented.^51^ Some papers also reported that the accuracy of responses was high for general or broad questions such as those on lifestyle, disease prevention, and health promotion.^39^,^48^,^52^,^53^

**Table 3:** Overview of Benefits and Risks Identified Associated with the Use of gAI/LLMs as Sources of Plain Language Information (N = 44)

Publication	What Worked Well with Respect to the LLM Assessed?	What did not Work with Respect to LLM Assessed?	Risks or Concerns of Using LLM	Recommendations (Related to Governance, Better LLM Model, Better Oversight, Self-Regulation, Standards, etc).
**Studies evaluating large language model**				
**Polat 2024**^22^	The AI model demonstrated successful performance on accuracy in all areas, including the “diagnosis and preparation process”, “surgical information”, “risks and complications”, and the “postoperative process”	ChatGPT’s answers may be too complex for some readers, as they are generally written at a high school level. This is above the sixth-grade reading level recommended for patient information by the AMA. The majority of the AI-generated responses were at or above the 10th-grade reading level, raising concerns about the text’s readability	Possible worries about bias, a lack of critical thinking, factual inaccuracies, and security concerns associated with ethical concerns, legal issues, privacy concerns, research fraud risk, and misinformation generation leading to infodemics	NA
**Sarraju 2023**^23^	The majority of ChatGPT’s responses to questions were graded as appropriate	ChatGPT’s responses to a few questions were graded as inappropriate, which could sometimes be incorrect and potentially harmful for certain patients	NA	NA
**Coban 2024**^24^	NA	NA	Ethical concerns may involve copyright infringements, medical and legal complexities, as well as the presence of misinformation or biases in the content	The role of healthcare professionals in information provision and contextualization could expand, becoming even more relevant than before, and AI language models could facilitate communication between healthcare service experts and patients
**Biswas 2023**^25^	The majority of ChatGPT responses were rated good or very good by the evaluators	A small proportion of the responses were inaccurate or flawed. Although ChatGPT shows an overall accuracy in its responses on myopia, it was limited by its inability to critically appraise/analyze results from the literature, knowledge database after 2021 (not updated), misinterpretation of medical terms, incapability to differentiate between predatory and reputable journal articles, and lack of scientific accuracy and reliability, biased and potential misinformation for readers	Lack of transparency on training and testing data used data bias, data abuse, and unreliable fact-checking. Little is known about the content creation, its origin, and weightage towards any industry or entity (a potential source of bias)	NA
**Balel 2023**^26^	ChatGPT provided reasonably accurate and helpful responses to patient-oriented questions	ChatGPT did not perform as well in responding to advanced technical questions	NA	NA
**Ghanem 2024**^27^	The majority of responses were graded as “accurate requiring minimal clarification” or “excellent”, and no answers were deemed inaccurate or harmful	NA	NA	NA
**Nielsen 2023**^28^	The chatbot exhibited the highest performance in the relevance category, followed by the accuracy	Responses lacked the depth of information and were challenged to understand and respond to complex queries that require a deep understanding of the context and the subject matter. There is a concern that Chatbot could spread misinformation as responses could be influenced by the biases contained in the data they were trained on	NA	NA
**Seth 2023**^29^	ChatGPT-4 provided well-structured, grammatically accurate, and comprehensive responses to the questions posed	ChatGPT was limited in providing personalized advice and sometimes generated inappropriate or outdated references	Negatively affect the doctor-patient relationship. Bias in information to mislead patients	Personalized responses need addressing in future models of ChatGPT if it is to be integrated into medical practice, especially considering the inability to provide specific, personalized advice goes against long-standing trends in medicine that seek to provide individualized and nuanced care
**Floyd 2024**^30^	NA	Chat GPT frequently generated inaccurate or incomplete responses missing essential context to patient-centered questions. When provided with the full-text article, it improved accuracy and comprehensiveness. During routine use, providing full text is not practical from the patient’s perspective	NA	In addition to patient education on these risks, it may be appropriate for natural language processing models such as ChatGPT to include a warning or risk statement when faced with medical inquiries, using language recommended by the Institute for Safe Medication Practices. The public is educated on the potential risks to patient health and safety associated with the use of this novel technology in the medical setting
**Keysser 2024**^31^	NA	The responses from ChatGPT lack sufficient scientific evidence. The nature of the question significantly influences the quality of statements. ChatGPT’s response was sensitive to “framing”. How questions were asked had an influence on the quality of recommendations.	NA	The uncritical use of ChatGPT as a patient education tool cannot be recommended at present
**Valentini 2024**^32^	ChatGPT’s responses scored better in the metric of appropriateness	Responses provided by ChatGPT to sarcoma-related questions were very inconsistent in quality, ranging from very good to very poor ones. Worst scores were observed in the accuracy domain	Sarcoma physicians should be aware of the risks of misinformation that ChatGPT poses and advise their patients accordingly	NA
**Rasmussen 2023**^33^	ChatGPT provided relevant responses to typical patient and parent questions	The study found that ChatGPT provides inaccurate and potentially dangerous statements, particularly regarding treatment and potential side effects of medications	NA	NA
**Studies comparing large language models with current standards (eg, established healthcare information or HCP)**				
**Rahimli Ocakoglu 2024**^51^	NA	NA	NA	NA
**Stroop 2023**^34^	ChatGPT provided good results in terms of comprehensibility, specificity, and satisfaction of responses and in terms of medical accuracy and completeness	In isolated cases, ChatGPT provided medically inaccurate claims, which raises serious concerns	The problem of how far patients can and may be informed using AI systems remains an ethically important point of discussion	LLM will not and must not replace medical communication between physicians and patients
**Citron 2023**^35^	NA	LLM generated automated but realistic-looking patient reviews and provided incorrect information about a surgeon’s CV. The source of information used to generate the response was opaque	There may be a blend of information from trusted and non-trusted sources, making the inaccuracies harder to detect. “In addition to collating existing information, the NLPTs can also generate new content. This was illustrated in the response to the questions about particular surgeons. The NLPTs collated an accurate CV but this was followed by fictional patient reviews generated by the bot. This raises ethical issues as the surgeon would not knowingly want to be represented by inaccurate reviews”	NA
**Currie 2023**^36^	LLM provided patient information that was largely considered fit for the purpose	The information provided by LLM lacked accuracy and currency and omitted important information	“Generalizations that could be misleading and errors, both of which threaten professionalism and the validity of informed consent”. “The shortcomings of GPT-3.5 are likely to increase the time demands on nuclear medicine staff for providing clarification and ameliorating any anxiety produced by discrepancies. These observations are counter to the purported benefits of AI generally, and ChatGPT specifically, in supporting patients and clinicians”	The value of GPT-3.5 in the patient information arena might be better targeted at translating existing patient information when English is a second language
**Sciberras 2024^a^**^37^	Overall, accuracy was high across all question groups	While ChatGPT has shown promise in producing mostly accurate and comprehensive responses, instances of either incomplete answers with no recommendations or incorrect answers have been noted. None of the answers contained links to the source of evidence to support the recommendations. A number of examples highlighting the limitations of ChatGPT were provided	NA	More clarity is also required in how these answers were formulated in terms of authorship, source, and date when the information was last updated. One potential strategy to enhance the reliability and personalization of information on these platforms could involve implementing a minimum requirement for medical information input from users
**Szczesniewski 2023**^38^	The overall information provided by ChatGPT was considered well-balanced or of moderate quality, varying across domains assessed	Chatbot does not disclose the sources of information and may contain bias even with simple questions related to the basics of urologic diseases	Has the potential to introduce biases by incorporating untruthful information from internet sources, which may contain a commercial component	Professional associations should engage with developers to ensure that accurate and tailored answers about common urological conditions are provided by AI in a clear and detailed manner, following the lines of action established for social media
**Gabriel 2023**^39^	ChatGPT’s responses generally contained accurate information, appropriate and pertinent to a patient’s potential inquiry, in line with the information the consultant urologists would provide to the patient in an outpatient setting	ChatGPT made a significant error while answering “What is the incidence of infertility after robotic radical prostatectomy?” claiming that “not all patients experience infertility after this procedure	NA	Strategies need to be developed by clinicians and clinical providers on the best way to incorporate these technologies, with the relevant oversight, to ensure their safest and optimum use for patients
**Walker 2023**^40^	ChatGPT provided low-to-moderate quality information comparable to available static internet information	One event of AI hallucination	ChatGPT does not specifically highlight medical advice that is contested or subject to debate. AI does not inform its users which medical information is controversial, which information is clearly evidence-based and backed by high-quality studies, and even which information represents the standard of care. Handling new and breakthrough information will also pose a major challenge for this application, as it is not able to understand the relevance of information per se but weights its importance based on previously available information, which could potentially be a detriment to new knowledge	Sources of medical information used by the AI software should be limited to peer-reviewed published data, and a bibliography should be implemented to allow for transparency of the provenance of information. The role of healthcare professionals in providing and contextualizing information may grow and become more relevant than ever, and AI language models might even facilitate communication between healthcare professionals and patients
**Cappellani 2024**^41^	ChatGPT is able to answer some questions correctly and completely as per the AAO patient guidelines	ChatGPT, on its own, provides incomplete, incorrect, and potentially harmful information about common ophthalmic conditions	NA	As the use of chatbots increases, human medical supervision of the reliability and accuracy of the information they provide will be essential to ensure patient’s proper understanding of their disease and prevent any potential harm to the patient’s health or well-being
**Casciato 2024**^42^	In terms of quality, ChatGPT maintained a rating of “good”, while FootCareMD was “excellent”	The overall readability of ChatGPT-produced responses was more difficult than that of human-produced patient information. Responses missed source/citations, disclosures, and currency (date of last update)	NA	NA
**Roldan‐Vasquez 2024**^43^	Surgeons unanimously found the ChatGPT’s responses understandable and actionable per the PEMAT criteria. ChatGPT acknowledged its informational role and did not attempt to replace medical advice or discourage users from seeking input from a healthcare professional	NA	Ethical concerns such as data privacy, security, transparency, accountability, bias, and fairness. Possibility of personal medical data being recorded and potentially used without explicit consent. Sourcing information from biased or unreliable sources which could negatively influence patients’ perceptions and decisions about their treatment	Vigilant evaluation of AI use in medical decision-making processes and the careful evaluation of AI use in sensitive medical context
**Janopaul-Naylor 2024**^44^	ChatGPT and Bing AI provided numerous cogent responses to common cancer patient questions	Serious or extensive shortcoming was noted by at least one panelist in 3% of chat GPT responses	NA	A critical need for continual refinement to limit misleading counseling, confusion, and emotional distress to patients and families
**Verran 2024**^45^	AI-generated PILs regarding medications were able to cover a number of key criteria, similar to PILs produced by the British Association of Dermatologists	AI-generated PILs were found to include similar factual content but excluded information that was felt to be more pertinent to patient concerns, such as curability and heritability. The readability of AI-generated PILs was beyond that of a large number of UK adults	NA	Caution is advised with regard to medication-specific patient information leaflets prior to distributing them to patients, owing to the risks associated with incomplete information and medication safety
**Abou-Abdallah 2024**^46^	NA	ChatGPT can simplify information at the expense of quality, resulting in shorter answers with important omissions. Limitations in knowledge and insight curb its reliability for healthcare information. ChatGPT’s ability to simplify information comes at the expense of content	NA	NA
**Halawani 2024**^47^	ChatGPT accuracy pertaining to kidney stone-related information showed an overall high accuracy with up-to-date information consistent with PIMs from international urological organization	AI model-generated responses were less readable than patient information material developed by the urologic organization	The chatbot’s inability to meet a prompt requesting a target reading level indicates a limitation of the technology, which may be related to the complexity of the source language used for its training	NA
**Lopez-Ubeda Valentini 2024**^48^	Summaries offered by the AI model are similar to those offered by the radiologist in all aspects. Participating radiologists agreed that the simplified reports are generally accurate and comprehensive and do not harm patients	Some cases of hallucinations, including non-relevant information, missing pertinent findings, poor section structuring, or errors in the writing format	Radiologists also identified incorrect statements and omissions of pertinent medical information in a significant number of these reports, which could lead to potentially detrimental summaries by patients	NA
**Lockie 2024^a^**^49^	The Chat GPT-generated PIL was assessed as being as good or slightly better than the surgeon-generated version	NA	NA	NA
**Coskun 2023**^50^	NA	ChatGPT produced a larger amount of information compared to the reference, and the accuracy and quality of the content were not optimal, with all scores indicating the need for improvement in the model’s performance	AI models are limited by the data they are trained on, and errors or biases in that data can lead to inaccuracies. There may also be ethical and legal concerns, particularly regarding privacy and data security. There is a potential risk of oversimplification in AI model-generated responses, with an example response that provided an incomplete/debatable statement	Much like the medical community, ChatGPT learns by supervised and unsupervised learning. Unlike the medical community, however, this platform learns at a pace we have never encountered
**Studies comparing different large language models**				
**Lim 2024**^54^	The overall language employed by Claude was professional yet avoided using excessive medical jargon. Claude’s guidance was competent; it was characterized as unexceptional	ChatGPT’s reply was generally broad, lacked details, and did not provide links or references to support its response. Bing provided hyperlinks for citation purposes and illustrative images for certain queries. However, the visual aids and several links were not helpful. Most of the links directed users to non-scholarly websites, undermining the credibility of the provided information	Surgeons may be hesitant to integrate AI-driven perioperative tools into their practices due to the potential legal liability from errors in judgment or delays in care caused by such AI technologies. The ethical integration of AI in surgical procedures raises significant concerns regarding privacy, consent, and human oversight	Maintaining human oversight is vital to ensure AI supplements rather than replaces professional medical judgment
**Hillmann 2024**^55^	Responses generated by AI LLMs were easy to understand. All questions encompassing clinically relevant decisions, all models recommended to consult the healthcare provider/physician	The study found that the appropriateness of information provided by AI was limited, and relevant content was often missing	NA	“The experts had to read carefully to detect slight but important misstatements in the given response”. This is an important point as patients would not be equipped to pick such details and may believe the answer is fully accurate, which might be misleading
**Ostrowska 2024**^56^	In the realm of symptoms and diagnostic inquiries, responses by LLMs were deemed safe and reliable by medical professionals	Novelties and upcoming treatment categories were the worst graded in quality and safety	NA	“The focus should not be on whether it is ethical for patients to use AI—since they will do so regardless—but on how we can guide them to use it responsibly”. Critical importance of oversight in using AI for medical guidance
**Patil 2024**^52^	ChatGPT and Bard generally provide accurate information regarding the risks, benefits, and alternatives in our provided CT and MRI scenarios	Due to the lack of detailed scientific reasoning and the inability to provide patient-specific information, both AI chatbots have limitations as patient information resources. Examples of incorrect information that is misleading and potentially harmful were provided	A pertinent drawback of AI chatbots was highlighted as the chatbots made assumptions about indications for the study, presence or absence of contrast, and patient characteristics such as age, sex, and comorbidities. Models are unable to provide personalized recommendations or ask for further information and, thus, are not well-suited for medical applications. Patient confidentiality and medicolegal issues may prevent the early widespread adoption of AI chatbots in a medical context	NA
**Moons 2024**^57^	Bard was successful in reducing the reading level of the sections from JAMA and Cochrane to that of sixth graders but omitted substantial amounts of content	ChatGPT could simplify written patient information materials and improve readability but could not achieve the desired sixth-grade level of reading proficiency	NA	NA
**Cheong 2024**^58^	No incorrect or dangerous information was identified in any of the generated responses from LLM	NA	The adoption of a large language model AI needs to be conducted with the consideration of patient data confidentiality. Both Google Bard and ChatGPT have highlighted that their human AI trainers may access generated conversations and advised against sharing sensitive information	The use of ChatGPT in electronic health record environments calls for robust regulations to preserve the confidentiality and security of patient information. This could encompass specific guidelines and measures to prevent unauthorized access or misuse of data by third parties in the form of data anonymization, encryption, and secure storage
**Yurdakurban 2023**^59^	Chatbots demonstrated high reliability and good quality	Readability of the responses was difficult and targeted individuals with a college-level education	NA	NA
**Coskun 2024**^60^	The results suggest that ChatGPT models show substantial potential for providing accurate and complete patient information	Examples flagged where ChatGPT provided incorrect medical information and suggested ongoing evaluation and refinement of models as AI advances to ensure the accuracy and quality of generated information	NA	NA
**Bellinger 2024**^61^	NA	NA	NA	NA
**Studies comparing LLM with Google search**				
**Mastrokostas 2024**^62^	Answers provided by GPT-4 were also associated with a higher Flesch-Kincaid grade level and lower Flesch Reading Ease score yet had a similar word count compared to Google. GPT-4 cited more reliable web sources than Google, which tended to rely on social media and medical practice websites	GPT-4, like its predecessor, may not be fully accessible to the average American adult, whose health literacy is at or below an eighth-grade level	NA	NA
**Van Bulck 2024**^53^	The ChatGPT-generated responses were generally considered to be trustworthy and valuable by the experts. Most experts did not think that the use of the information provided by ChatGPT on the prompts was dangerous	The most common negative feedback was that certain information was missing, too vague, a bit misleading, and not written in a patient-centered way. The experts also recognized that the responses are often incomplete and sometimes misleading	NA	NA
**Ayoub 2024**^63^	ChatGPT scored higher with patient education questions compared to Google search	ChatGPT fared worse when responding to questions seeking medical recommendations and guidance compared to Google search	Because this technology has not yet been rigorously shown to be a safe or appropriate resource for patient education, healthcare professionals should consider whether there are any potential legal ramifications to recommending ChatGPT to patients	ChatGPT has the potential to improve patient-clinician communication and, in turn, reduce healthcare disparities. Because ChatGPT is blind to users, unconscious bias is theoretically eliminated, and no assumptions regarding health literacy levels are made. When elicited, ChatGPT can provide material at specific knowledge levels. Rapid transfer of medical knowledge in multiple languages can potentially decrease health disparities, minimize knowledge gaps, and improve informed decision-making
**Tharakan 2024**^64^	ChatGPT is more likely than Google to answer patient questions about TSA and TEA with academic sources	NA	NA	Patient autonomy
**Studies comparing AI app-based recommendations**				
**Griefahn 2024**^65^	Based on study results, the use of AI to provide exercise advice can initially be considered safe, which means that a large number of people with MSDs can be reached	In a substantial number of cases, at least one exercise was rated by at least one physiotherapist as being a risk to the specific patient example, raising concern about patient safety	NA	NA

**Note**: ^a^Involved actual patients in the study.

**Abbreviations**: AAO, American Academy of Ophthalmology; AI, artificial intelligence; AMA, American Medical Association; CV, curriculum vitae; gAI, generative AI; LLM large language model; JAMA, Journal of the American Medical Association; MSD, musculoskeletal disorders; NA, not applicable, NLPT, natural language processing tools; PEMAT, Patient Education Material Assessment Tool; PIL, patient information leaflet; PIM, patient information materials; TSA, total shoulder arthroplasty; TEA, total elbow arthroplasty.

The most common risks of using gAI/LLMs as a source of plain language medical information included concerns about the low readability of responses generated versus the requirements of the intended audience and oversimplification of responses at the expense of depth of information. Some papers also reported a decline in accuracy and completeness progressively with more specific or complex questions around disease symptoms, diagnosis, side effects of treatment options, and a lack of transparency on information sources used to generate responses.^28^ Some reported risks related to responses containing misinformation or inaccurate and outdated information and the absence of the ability to flag controversial or commercially biased information. There were also risks associated with incorrect generalization or extrapolation from source information and the blending of information from correct and incorrect sources. All of these could lead to errors or even harm to patients, which would require remediation and add to the burden of HCPs and healthcare systems. Another potential risk highlighted included data privacy and security concerns if patients start uploading personal medical data to ask gAI/LLMs for explanations. A contrasting risk could be that, in the absence of patient demographic data and unknown to the patients, the models may assume these parameters and provide responses that are not personalized.

None of the published primary research identified from our literature search actively commented on the ethical considerations of their findings.

### Recommendations for Implementing gAI/LLMs as Sources of Medical Information for Non-Specialist Audiences

A few papers included brief recommendations on how to de-risk the use of gAI/LLMs by non-specialist audiences (Figure 1). Model development or co-design should involve patients, the general public and HCPs to make responses more relevant to the audience at the time of implementation. This also allows the opportunity to integrate experience, awareness and diversity.^66^ Optimizing output from gAI/LLMs for better accessibility and understandability can be done through language translation options, complementing text with visuals, personalized output by implementing a minimum input requirement from patients, and incorporating a reinforced learning approach based on human feedback to fine-tune their conversational interaction quality. The reliability of output from gAI/LLMs can be improved by linking responses to peer-reviewed publications supported by a bibliography. Currently, available disclaimer statements warning users about the limitations of the models to offer medical advice should be enhanced to include a risk statement in line with safe medical practice recommendations. Responsible utilization of gAI/LLMs will require medical societies to stay vigilant, and HCPs could provide oversight and evaluate and contextualize the responses these models generate in a medical context.^66^ Including patients and the general public during the gAI/LLMs’ development phase could enable awareness and education, help avoid misconceptions, reduce fear, increase trust and acceptance of these models, and encourage responsible usage.^66^ However, patients and the public will have to be continuously educated about the appropriate use of gAI/LLMs. Finally, robust regulations will have to be implemented to preserve patient information confidentiality.

## Ethical Considerations

In this section, we provide a snapshot of the ethical considerations surrounding the use of gAI/LLMs by non-specialist audiences. This is based on a supplementary review carried out after our literature search, which found no primary research discussing ethically relevant content (summarized in Figure 1).

### Improving Patient Health Literacy, Enabling Empowerment and Shared Decision-Making (Respect for Autonomy)

One of the positive implications of gAI/LLMs discussed is their potential to improve patient health literacy and digital literacy by providing easily accessible and understandable medical information. This is particularly beneficial for those at lower health literacy levels and can have an overall positive impact on patient-HCP communication.^23^ These models can also support spreading disease awareness among the general public. gAI/LLMs offering visual graphical or audio-visual elements in their responses could enhance the educational value of their output for patients. These models also bring language translation opportunities, significantly improving access to medical information for non-native English speakers. Thus, gAI/LLMs may improve informed decision-making and reduce disparity in favor of patients in countries where English is a second language. Freely accessible, reliable medical information from gAI/LLMs can help those without the financial ability to seek immediate medical attention, thus reducing the risk of poor outcomes. If gAI/LLMs provide responses based on citable peer-reviewed literature, this access to reliable medical information can enable patient autonomy and informed decision-making.^66^ Taken together, these opportunities that gAI/LLMs bring support the first principle of biomedical ethics, ie, respect for autonomy (patients making informed, competent, independent decisions).

### Risk of Perception as “Quasi-Experts” (Principles of Nonmaleficence and Beneficence)

What is potentially the biggest epistemic concern is the projection of “quasi-humanness” by the general public onto gAI/LLMs. The tone of responses from gAI/LLMs has sometimes been rated as more empathetic and preferred to that of HCPs.^28^ This perception could distract users from verifying the reliability of the responses and encourage them to trust biased medical information. The other concerns are the lack of transparency about their source and the inability of the models to flag any controversial or unreliable information.^28^ In some cases, the bias in responses may also be commercial in nature. Furthermore, unlike traditional search engines, which provide a list of sources along with explanations, gAI/LLMs provide a single response that could be misconstrued. And, finally, the responses from these models vary in depth and accuracy depending on the framing of the query or prompt. This shortcoming is concerning, given that not all users can be expected to be at the appropriate level of health literacy or digital literacy. The two biggest unaddressed questions are, first, applicability – was this type of end-use considered during the development phase of these models and deemed appropriate? Second, there is a conflict of interest: who benefits financially from the models’ deployment? These concerns pose a risk to the second and third principles of biomedical ethics, ie, principles of nonmaleficence (to not intentionally harm patients by imposing careless risk) and beneficence (to be of benefit to patients or remove harm).

### Unclear Accountability for Outcomes/Decisions and Impact on Patient–HCP Relationships (Principle of Beneficence)

Another epistemic cum normative concern is the assignment of accountability for any harm or negative health effects or delays in seeking treatment or undermining medical advice due to decisions of patients based on responses from gAI/LLMs. This is a crucial unanswered question: Who provided expert consultation for the models, and who bears responsibility for the responses generated by gAI/LLMs and their impact? Unanticipated patient outcomes from unreliable responses may also add to the existing workload of HCPs and negatively affect patient-HCP relationships. HCPs who are already challenged for time may have to assume additional supervisory responsibilities verifying medical information and advice that their patients have received from gAI/LLMs. Whether there are any legal ramifications of HCPs guiding patients on how to use gAI/LLMs responsibly also needs consideration. These concerns may act as barriers to HCPs delivering on their duty towards patients through the third principle of biomedical ethics, ie, the principle of beneficence (to be of benefit to patients or remove harm).

### Risk to Equity, Inclusiveness and Data Privacy (Principle of Justice)

One of the normative concerns with gAI/LLMs is that bias in the source or training datasets due to a lack of diversity in patient demographics, medical conditions, and healthcare practices across institutions could creep into the responses and may not be generalizable. The unaddressed question here is whether different geographical and cultural contexts were considered and incorporated during the development of the gAI/LLM. The reading levels of the responses gAI/LLMs currently generate are very often at a higher level than the levels recommended for the general public. This puts those at a low health literacy level at a perpetual disadvantage.^51^ In their efforts to make complex topics readable, gAI/LLMs often omit important content and oversimplify their responses. Most of the latest versions of gAI/LLMs, which claim higher accuracy and the ability to tailor responses to individual needs, are subscription-based. This automatically introduces a barrier to equitable access to medical information and may introduce bias due to a limited user base and their interaction history. gAI/LLMs also harbor the risk of perpetuating equity-averse information, which could be exacerbated in the future. There is also a high risk that patients might upload personal medical records and other confidential information, which brings up significant concerns about data privacy and data security and the lack of informed consent.^66^ These concerns may pose a risk to the final principle of biomedical ethics, ie, the principle of justice (to distribute healthcare fairly).

## Ethical Basis for Implementing gAI/LLMs as Sources of Medical Information for Non-Specialist Audiences

In Table 4, we provide ethically deliberated recommendations from the literature and from our two patient authors (TB and MAR) for “safe” use of gAI/LLMs by non-specialist audiences. The overarching themes are: incorporate moral codes within the models so that those are reflected in the responses; integrate health equity domains into the implementation framework of gAI/LLMs; implement regulations, both self-regulation by developers and oversight by regulators; and, involve stakeholders in designing, developing, implementing, and verifying output.

**Table 4:** Ethically Deliberated Recommendations for “Safe” Use of gAI/LLMs by Non-Specialist Audiences

	General Recommendations	Patient Recommendations
**Incorporate moral codes**	Incorporate a common universal moral code and common moral philosophical concepts for the responses to reflect those values. If the models conform to consequentialist theories such as utilitarianism, which posits “the greatest happiness for the greatest number of people”, and egoism, which posits “do well by doing good”, they are most likely to generate outputs that are reliable and trustworthy for a broad audience.	To avoid prioritizing the majority group preferences over marginalized populations, the models can factor in non-consequentialist theories such as Kant’s theory of moral absolutism and Rawl’s theory of justice.^67^ The models could incorporate the theory of absolutism as a “universal rule” to “behave ethically” by generating responses that prioritize the needs of the audience.^67^
**Integrate health equity domains**	Accountability should rest with a governance committee within the healthcare delivery system, which has a clear understanding of the legal and inherent limitations of the models and can implement mechanisms of ongoing monitoring, feedback, and evaluation.^19^ For fairness, establish and evaluate criteria for the performance of the models to track progress in achieving health equity and identify the factors that unduly influence disparities. For fitness for purpose, define the target population and sustainability assessment to ensure the models meet the requirements and preferences of the intended users. For reliability and validity, implement ongoing assessments to identify whether the pre-specified goals and performance measures are being met. For transparency, actively engage in communication between stakeholders.^68^	Developers of these models should actively involve patients and HCPs in the development and validation process. Training in the use of these models should be provided for non-specialist audiences The developers of these models also need to be trained on patient engagement and how to involve end-users in the development phase in accordance with good patient engagement practices.
**Implement regulations**	Models must adhere to existing regulations such as the FDA’s Good Machine Learning Practice for non-LLM AI tools, Article 14 of the EU Artificial Intelligence Act and the WHO guidance on Ethics and Governance of Artificial Intelligence for Health.^20^,^69–71^ Developers should publish more clinical validation data and prioritize prospective studies.^4^ Independent third parties should audit and certify that these models meet certain transparency and ethical standards.^69–75^ Developers could consider designing a second layer of a “bias detective gAI/LLM”, which will verify the output generated by the first gAI/LLM against a valid information source.^76^ If feasible, this should be complemented by periodic random human-led quality control.	The onus of obligatory checking of output from the models should rest with the developers. Caution will be needed to avoid excessively rigid regulations, with the aim of protecting health and human rights, that may throttle innovation in.^77–79^ Developers can refer to upcoming recommendations from the International Society for Medical Publication Professionals,^80^ the UK’s Patient Information Forum^81^ and the Coalition for Health AI.^82^
**Involve stakeholders**	By engaging HCPs in the development and deployment of these models, developers can ensure that the models align with clinical best practices and prioritize patient well-being.^19^ HCPs need to be educated on the advantages and pitfalls of using gAI/LLMs in generating medical information. HCPs have to be the safety monitors, moral agents, and overseers of ethical and responsible adoption. An ideal scenario would be that at diagnosis, the HCPs encourage self-research but also forewarn patients and caregivers about the pitfalls and limitations of gAI/LLMs, provide reliable educational resources to them in their preferred format, as well as links to patient support groups or charities. This will ensure that the patients and caregivers can participate in shared decision-making.^73^,^75^	Develop a public registry of medical AI systems, including patient-facing gAI/LLMs, to improve “algorithmic literacy” among the general public as a fundamental precursor for human and legal rights.^83^ Develop models purely to address medical information queries from non-specialist audiences. Reduce the risk of hallucinations by implementing response verification mechanisms through close collaboration between a developer, publishers of peer-reviewed journals, HCPs, and global healthcare systems regulators.

**Abbreviations**: AI, artificial intelligence; EU, European Union; FDA, Food and Drug Administration; gAI, generative AI; HCP, healthcare professional LLM, large language model; WHO, World Health Organization.

## Discussion

This study is uniquely positioned as it brings together, first, a detailed review of the performance of gAI/LLMs as sources of plain language medical information for non-specialist audiences and, second, a critique of ethical considerations related to this application of gAI/LLMs. The critique is especially important because gAI/LLMs developers may not have envisioned the use of these models as a source of plain language medical information. This opens up these models to the fallacy of “discrimination from design”, which can undermine the accuracy of their output. Finally, we also provide ethically deliberated recommendations that may help make gAI/LLMs suitable for use by non-specialist audiences.

gAI/LLMs have been reported to provide responses that were, in general, accurate, easy to understand, and helpful to non-specialist audiences. This can enable these users to access, generate, and personalize information in ways that extend beyond the limitations of human cognition. Moreover, the conversational nature of LLMs and their “confident” tone of output enable these models to engage in an interactive dialogue with users who have the impression of chatting with a “quasi-human”.^2^,^84–86^ Such “anthropomorphization” or projecting human-like characteristics, behaviors, or intentionality to gAI/LLMs may build unwarranted trust and distract non-specialist audiences^28^ from “hallucinations” or “botshit”.^87^ This phenomenon may negatively influence patient safety and fuel misconceptions and misinformation due to overgeneralized or extrapolated gAI/LLM-developed conclusions.^2^,^84–86^ Furthermore, while the conversational nature improves the ease with which the models interpret and understand information via “informational transparency”, on the other hand, the lack of “reflective transparency” creates opacity in the data source and algorithmic process, and does not allow non-scientific audiences to assess biases in the model and understand the reliability of the recommendations. Given the high usage of internet search engines, the majority of the general public either already are or will be exposed to gAI/LLMs-generated plain language content online, even if they do not access the gAI/LLMs directly. Patients also expect transparency regarding whether they are accessing gAI/LLMs or traditional search engines such as Google or Bing while searching for medical information, as these are often co-located and confusing to those who may not be at an appropriate digital literacy level. They also expect transparency from patient organizations to declare when gAI/LLMs are used to create plain language materials and what policies are in place for appropriate safeguarding of the output. Most available gAI/LLMs caution users not to use their responses for medical information, diagnosis, or decision-making. However, it is not a sufficient deterrent, and real-time monitoring is not realistic. In this context, there are significant concerns that gAI/LLMs often act as “black boxes”, which makes it difficult for non-scientific audiences to interpret the reasoning process that leads to the responses. Recent efforts have focused on establishing standards of assessment for responses to medical questions. However, these efforts still do not include the perspective of non-specialist audiences.^88^ This unmet need for better explainability requires urgent attention in order to build the non-specialist audiences’ trust in the transparency and accuracy of these models.^89^

There is potential for gAI/LLMs to improve health literacy and enable non-specialist audiences to participate in shared decision-making. However, in this context, the assignment of accountability for outcomes, especially potential harms from responses of gAI/LLMs, is a key question to consider. This is crucial, given that the output that these models provide is probabilistic and varies depending on prompts. The models may also exhibit “sycophancy bias” – a tendency to tailor their responses to perceived user expectations – leading to incorrect confirmation bias coupled with pseudo-confidence.^84^,^90^ Issues surrounding data quality, potential biases, the opaque nature of the algorithm-generated information and the involvement of multiple stakeholders in their development and implementation complicate the assignment of accountability.^91^,^92^ Furthermore, as these models are iterative with shifting goals and because they continuously learn new patterns, it will be challenging to direct the liability of any adverse outcome to the technology developers.^85^,^93^ Given the complex nature of the stakeholder matrix involved in the development and implementation of these models, this lacunae of assigning accountability is concerning and bears significant risk to the well-being of non-specialist audiences. A related theme that requires further consideration is how significantly gAI/LLMs could exacerbate and perpetuate social inequalities if access to these models is prioritized only for developed geographies.

The erosion of the “human connect” and a change in the patient–physician relationship could be another potential fallout of the direct use of gAI/LLMs by non-specialist audiences.^93^,^94^ When these models become an alternative source of plain language medical information that patients use to challenge diagnosis or treatment plans, to verify HCPs’ recommendations, or as an alternative to remote consultations and long waiting lists, they could have long-lasting impacts on the patient-HCP relationships.^5^,^7^,^85^,^93^ Patients have also reported using gAI/LLMs to largely derive positive outcomes during their interactions with healthcare systems. This may create an illusion of lowering dependence on HCPs for healthcare decision-making. They have used these models to organize medical records in preparation for their HCP consultations, to comprehend medical records and literature, to assist in the diagnosis of complex rare diseases that remained undetected by multiple specialists, to correct misdiagnosis, and to analyze symptoms while managing long waiting times for HCP consultations.^5^ However, patients continue to see an important role for HCPs in ensuring optimal disease management and overseeing safety aspects as a safeguard while using gAI/LLMs for medical information.^85^ The encounter between a patient and HCP is conceptualized as an opportunity for “co-reasoning”, leading to a reasoned decision. In a role reversal, a potential area for future investigation could be whether this construct is challenged depending on how patients perceive the use of gAI/LLMs by HCPs in decision-making and care delivery.^95^

Our exploration of this topic is an attempt to direct the attention of the various stakeholders to this almost overlooked use of gAI/LLMs by non-specialist audiences to obtain plain language medical information. While there are opportunities, this usage comes with significant risks in the current environment of minimal regulation or supervision of both the models and their users. We sincerely hope that the risks and ethical implications highlighted in our paper encourage HCPs and policymakers to critically assess and implement regulatory frameworks to holistically enable “safe” use of gAI/LLMs by patients, caregivers, and the general public. The recommendations from our patient authors are particularly relevant in this context, as they bring valuable but often ignored patient voices into this conversation.

## Conclusions

The use of gAI/LLMs will shape how non-specialist audiences navigate healthcare systems in the future. Access to reliable, understandable and personalized medical information through gAI/LLMs can empower non-specialist audiences, improve health literacy and enable informed decision-making and active participation in their self-management. Furthermore, gAI/LLMs have the potential to address health disparities by providing culturally sensitive health information and language support for a diverse population of patients who are not being sufficiently served by the public health systems. However, as we note, these opportunities are accompanied by significant cause for concern. Currently, there is a lack of sufficient oversight, regulation, and training on the development and use of gAI/LLMs by non-specialist audiences to obtain plain language medical information. In addition, there is a need to effectively address the ethical concerns related to explainability and accountability to maximize positive outcomes for all stakeholders. We recommend that for gAI/LLMs to be truly transformational sources of plain language medical information, they need to be more transparent in their algorithmic functionality, undergo appropriate and continuous governance and monitoring, and have mechanisms in place for improvement through feedback and input from patients, HCPs, and other experts.
